# The uses of *Chrysomya megacephala* (Fabricius, 1794) (Diptera: Calliphoridae) in forensic entomology

**DOI:** 10.1080/20961790.2018.1426136

**Published:** 2018-03-21

**Authors:** Rozane Badenhorst, Martin H. Villet

**Affiliations:** Southern African Forensic Entomology Research Laboratory, Department of Zoology and Entomology, Rhodes University, Grahamstown, South Africa

**Keywords:** Forensic sciences, forensic entomology, *Chrysomya megacephala*, morphology, taxonomy, biogeography, life cycle, ecology

## Abstract

*Chrysomya megacephala* (Fabricius, 1794) occurs on every continent and is closely associated with carrion and decaying material in human environments. Its abilities to find dead bodies and carry pathogens give it a prominence in human affairs that may involve prosecution or litigation, and therefore forensic entomologists. The identification, geographical distribution and biology of the species are reviewed to provide a background for approaches that four branches of forensic entomology (urban, stored-product, medico-criminal and environmental) might take to investigations involving this fly.

## Introduction

The blow fly *Chrysomya megacephala* (Fabricius, 1784), commonly called the oriental latrine fly [1], has significance for public health, food industries, medical entomology, investigations of deaths and, most recently, industrial recycling of organic waste. All of these concerns may become the subject of prosecution or litigation, and can therefore involve forensic entomology. Large populations of *C. megacephala* may inhabit human settlements, and adult flies visit moist foodstuffs and decaying carrion and faeces, where they may breed very abundantly. To compound its significance, this synanthropic species has spread almost globally from its native geographical distribution. The wide range and global ubiquity of human concerns associated with *C. megacephala* merits a review of the species’ biology as a foundation for research into its management and applications, particularly for forensic entomology. Therefore, this paper reviews published literature about this species in relation to each area of concern; a comprehensive bibliography is beyond the scope of this review.

## Taxonomy

Correct identification of an organism is a cornerstone of forensic entomology, primarily because it facilitates access to relevant information stored in published literature and allows scientists to communicate and contextualize their findings effectively. *Chrysomya megacephala* is a case in point: it has 15 primary synonyms (Table 1), 3 putative forms and a history of being confused with other members of the *megacephala* species-group in scientific publications.

**Table 1.** Primary synonyms of *Chrysomya megacephala* (Fabricius, 1794) (from Pape and Thompson 1915).

No	Synonym	Taxonomic author
1	*Musca megacephala*	Fabricius, 1794: 317
2	*Musca dux*	Eschscholtz, 1822: 114
3	*Chrysomya duvaucelii*	Robineau-Desvoidy, 1830: 451
4	*Chrysomya gratiosa*	Robineau-Desvoidy, 1830: 451
5	*Lucilia flaviceps*	Macquart, 1843: 302
6	*Musca flaviceps*	Walker, 1849: 870
7	*Musca remuria*	Walker, 1849: 871
8	*Musca bata*	Walker, 1849: 875
9	*Musca combrea*	Walker, 1849: 876
10	*Pollenia basalis*	Smith, 1876: 449
11	*Somomya pfefferi*	Bigot, 1877: 257
12	*Somomyia saffranea*	Bigot, 1877: 257
13	*Somomyia dives*	Bigot, 1888: 600
14	*Somomyia cyaneocincta*	Bigot, 1888: 604
15	*Somomya cyaneocincta*	Bigot, 1888: 604

The species was divided into three morphological forms with distinct ecologies [2,3]: a “normal” southern Pacific, forest-dwelling form with relatively small dorsal ommatidia in the male; a “derived”, synanthropic and now cosmopolitan form with relatively large dorsal ommatidia in the male; and a “feral derived” Himalayan form with intermediately sized dorsal ommatidia in the male [3]. However, the “normal” form was subsequently described as *Chrysomya pacifica* Kurahashi, 1991 [4] and molecular evidence suggests that it is a distinct species [5]. The status of the feral derived form awaits molecular evidence, but seems likely on morphological grounds to be an independent species too, cf. [3].

In older publications, *C. megacephala* was notably confused with the closely related *Chrysomya saffranea* (Bigot, 1877) [6] and the less-closely related *Chrysomya bezziana* Villeneuve, 1914 [1,7], both of which belong to the *megacephala* clade or species-group [5]. Both of these species may occur in forensic cases involving death or neglect of people or animals. Care should be taken to verify the taxonomy of older studies before using their findings. Kurahashi's [2] *defixa*-subgroup appears to be a grade defined by plesiomorphic characters [2,5], and should be included in the *megacephala*-group clade.

### Morphological identification

#### Adult

**Figure 1.**
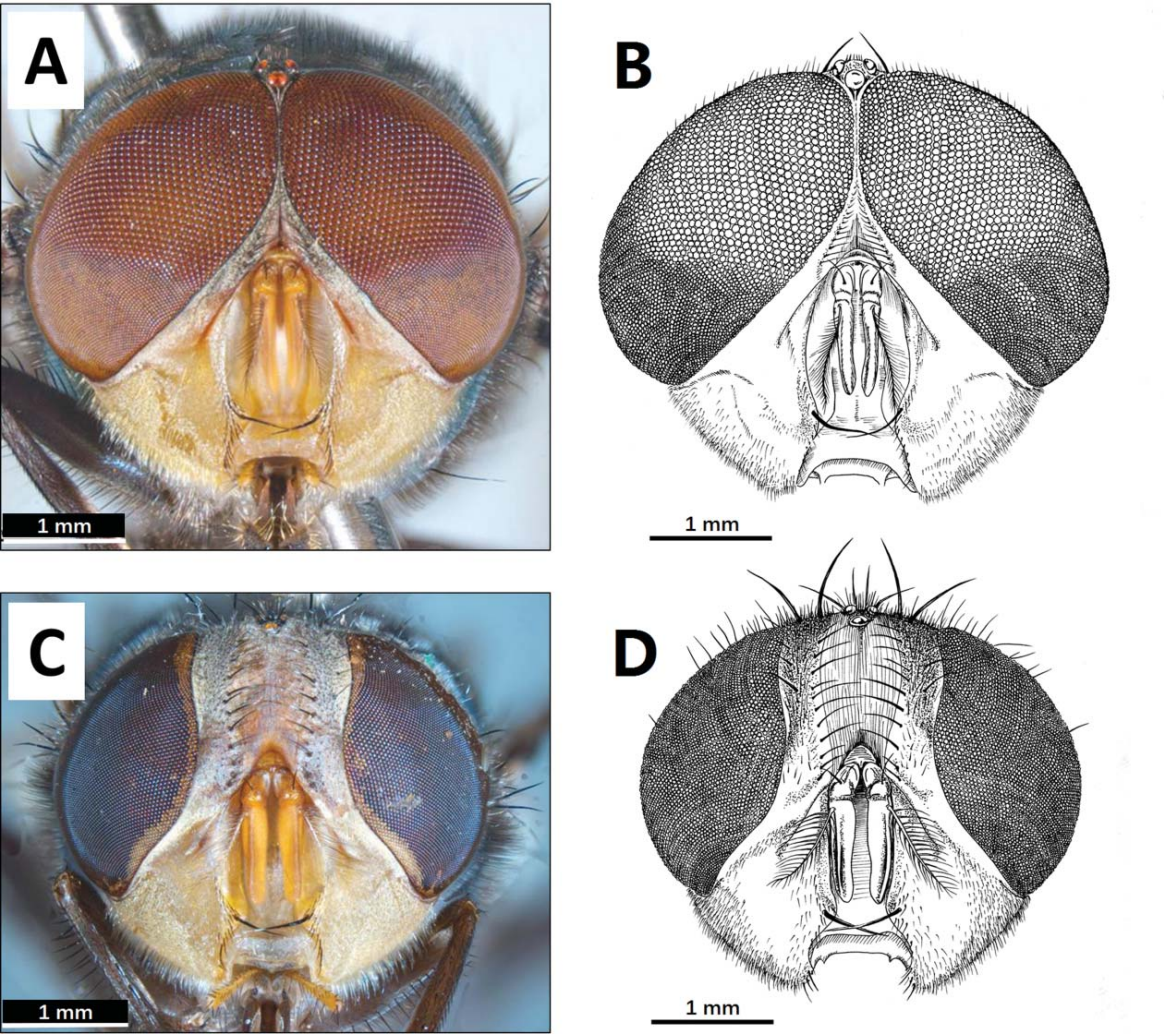
Anterior view of the head of a male (A and B) and female (C and D) adult of *Chrysomya megacephala*, showing the sharply differentiated sizes of the upper and lower facets of the male's eyes, and the shape of the female's frons, which has convex sides. Images A and C by Ken Walker via PaDIL - http://www.padil.gov.au under the Creative Commons Attribution 3.0 Australia License.

A morphological identification key to adults, including the named species of the *megacephala* species-group, has been published [8]. The adults of *C. megacephala* have been illustrated with drawings [1,9–11] and photographs [12–14]. The differentiation of males’ eye facets (Figure 1(A,B)), the shape of females’ fronses (Figure 1(C,D)) and the colours of the anterior thoracic spiracle and the lower calypter are particularly important in the morphological differentiation of this species from its close relatives [1,8]. The diagnostic males’ genitalia are illustrated by Zumpt [1] and Chaiwong et al. [15], and the females’ by Bansal and Murad [16].

#### Spermatid

Comparative studies that would aid the identification of spermatozoa of *C. megacephala* have not been published. The spermatozoa are typically brachyceran, about 0.6 mm long and very thin; the head forms about 10% of the total length [17].

#### Egg

Comparative studies that would aid identification of the eggs of *C. megacephala* have not been published. The eggs [1] are 1.5–1.6 mm long, sausage-shaped and whitish, turning cream. Unlike the eggs of other blow flies [18], and particularly the closely related *C. pacifica* [19], they are reported to have a wide, flattened strip running the length of one side that bears leaf-like projections [20]. If these observations are correct, the projections would be taxonomically diagnostic.

#### Larva

The larvae have been illustrated with drawings and photographs, and there are studies of the ultrastructure of the larval exoskeleton [21–24]. Identification usually involves the mouthparts, the anterior spiracle and the bands of spines on each segment.

First-instar larvae [1,21,24] are 1.7–3.5 mm long, and the posterior spiracles have one slit. Keys are available that differentiate the first-instar larva of this species from many blow flies from Africa and Europe [24,25].

Second-instar larvae [1,21] are 6–8 mm long, and the posterior spiracles have two slits. There is no specific identification key for this instar, which differs slightly in morphology (particularly its mouthparts) from the first and third instar.

Third-instar larvae [1,21,26,27] grow to about 16 mm long, depending on the temperatures they experience, but contract rapidly just before pupariation [28], and the posterior spiracles have three slits. The band of spinules on the last segment is incomplete dorsally. The anterior spiracle has 11–13 branches.

#### Puparium

The puparium is formed from the exoskeleton of the third-instar larva, and thus bears the same identifying surface structures as the mature larva. It is brown with yellow anterior spiracles, as in most blow flies [1,24,29,30]. The mouth hooks of the third-instar larva, which can assist identification of pupae, can usually be found adhering to the inside of the eclosed puparium.

### Molecular identification

DNA sequences can identify samples, but the choice of marker molecule is important. Mitochondrial DNA (mtDNA) markers evolve four times faster than nuclear DNA markers and are present in many times more copies per cell; they are therefore preferred for identifying species. However, mtDNA can fail to distinguish closely related species under some conditions, including recent hybridization and incomplete lineage sorting [31]. Introgression (ancient hybridization) may also pose problems for forensic entomology, especially if the species has not been studied before [31,32].

The whole mitochondrial genome of *C. megacephala* has been sequenced [33]. The mitochondrial control region could be used to identify conspecific specimens in phylogenetic analyses, but can be difficult to align consistently, which creates problems for identification by standard phylogenetic methods [34]. Dynamic homology analysis (implemented in, e.g. POY: [35]) or alignment-free analysis (implemented in, e.g. kSNP: [36]) may resolve this issue in a future study.

The popular DNA “barcode” (∼670 bp of the 5’ end of the mitochondrial *cytochrome c oxidase subunit I* [*COI*]) generally succeeds as an identification tool for blow flies [37–42], and benchmark sequences for *C. megacephala* are available on the National Center of Biotechnology Information and Barcode of Life databases (GenBank and BOLD, respectively). Phylogenetic analysis of *COI* sequences from adult specimens of *C. saffranea* and *C. megacephala* indicated hybridization and perhaps also incomplete lineage sorting [38,39]. Similar results were obtained from analysis of a 2 200 bp sequence encompassing the barcode fragment of *COI* and the *cytochrome c oxidase subunit II* (*COII*) and *t-RNA leucine* genes from larvae of *Chrysomya pinguis* (Walker, 1858) and *C. megacephala* [43]. Even whole mtDNA sequences might not provide unequivocal differentiation of *C. megacephala* from its near relatives when lineage sorting is far from complete. “Barcoding” of forensically important insects also has a surprisingly high failure rate for technical reasons [39–42,44]. “Barcoding” is problematic for forensics when there is no means to cross-validate an identification [45,46].

Because nuclear markers evolve at a quarter of the rate of mtDNA, they are not expected to perform better under incomplete lineage sorting. An example is that the nuclear *bicoid* gene shows paraphyly between *C. megacephala* and *C. pinguis* [32] that might be due to introgression.

### Phylogenetic relationships

The more closely related two species are, the more likely they are to be confused in forensics investigations (and to hybridize naturally), and these species pairs can be identified by phylogenetic analysis. Phylogenetic analyses of DNA sequences of the mitochondrial *COI* and nuclear *carbamoylphosphate synthetase* genes showed that the probable sister species of *C. megacephala* is *C. pacifica*, and that this species pair is (increasingly distantly) related to *Chrysomya cabrerai* Kurahashi & Salazar, 1977*, Chrysomya defixa* (Walker, 1856), *Chrysomya greenbergi* Wells & Kurahashi, 1996, *C. pinguis*, *Chrysomya thanomthini* Kurahashi & Tumrasvin, 1977, *C. bezziana*, and *Chrysomya chani* Kurahashi, 1979 [5]. The exact relationship between *C. megacephala* and *C. saffranea* has not been established, but the hybridization and perhaps incomplete lineage sorting between them [38,39] indicates that they are about as close as *C. megacephala* and *C. pinguis* [43].

Several of these species are known to breed in carrion and can be quite common, emphasizing the need for correct identification in forensic entomology. All of these species inhabit the Oriental and Australasian Regions.

## Biogeographical distribution

The original distribution of *C. megacephala* was in the Oriental and Australasian Regions where its close relatives occur; its closest relative, *C. pacifica*, inhabits the islands between New Guinea, Samoa and New Caledonia [2,4]. *Chrysomya megacephala* also occurs in the Indian Region and the Middle East [47]. Two of its synonyms, *Pollenia basalis* Smith, 1876 and *Somomyia pfefferi* Bigot, 1877, were described from the islands of Rodriguez and Mauritius, respectively [48,49], implying that the species was present there by the dates of those descriptions. Fabricius [50] gives the type locality of *Musca megacephala* as “Guinea” and the donor as “Dr Ifert”, who collected in the Danish Gold Coast (or, in Danish, *Dansk Guinea*), approximately in modern coastal Ghana (Thomas Pape, personal communication). These details imply that *C. megacephala* may in fact have occurred in West Africa before 1794, which would affect the interpretation of its historical geography.

**Figure 2.**
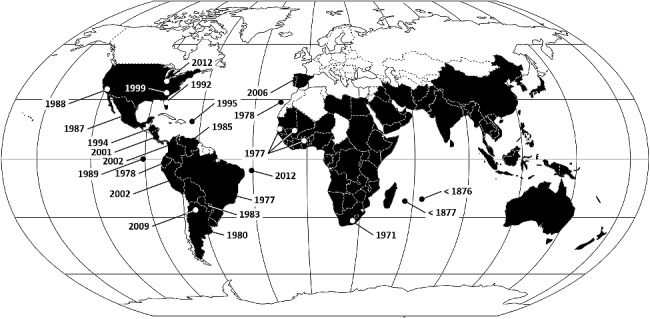
Geographical distribution of *Chrysomya megacephala*, by country, with year of discovery outside native range (after [14,47,51,52,58,60–63]).

*Chrysomya megacephala* has spread dramatically through Africa and the New World (Figure 2). It was first found on the African mainland in South Africa in 1971 [51] and (re)discovered abundantly in West Africa in 1977 [52]. Although it has invaded much of sub-Saharan Africa, it is largely absent from Asia Minor and Europe, except for Spain [53], Malta [54] and Portugal [55]. The earliest Neotropical locality records are from south-eastern Brazil in 1977 [56,57]. It was collected in North America at Baja California, Mexico, in 1987 and Florida in 1992 and appeared in Indiana, USA, in 2012 [58]. There is genetic evidence that the Florida population is drawn from two sources [59]. This species has also reached isolated oceanic localities like the Galapagos and Fernando de Noronha archipelagos [60] (Figure 2). Although it has not been reported from Morocco, Western Sahara, Chad, the Guianas and Chile, it probably occurs in those countries and therefore throughout sub-Saharan Africa and South America.

New geographical appearances of pestilent blow flies quickly draw attention [7,10,53–56,58,60,64–70], allowing rough estimates of the rate of spread of the species. The species apparently arrived irruptively in Curitiba in 1976 [56] and spread promptly through coastal Brazil [57]. Recent Argentinian records of *C. megacephala* extend westward by approximately 500 km from the known distribution of the species [70]. Since blow flies are estimated to travel 2-3 km/day [71,72], such dispersal need not rely on human transport [51].

*Chrysomya megacephala* clearly inhabits tropical and temperate climates (Figure 2), although it was reported from Eastern Siberia, at about 50°N [73]. Although commonly termed synanthropic, it also occurs naturally in areas with little human presence [60]. With current trends in global climate, it seems likely that *C. megacephala* will colonize Europe and Canada, and therefore occur throughout the planet wherever climates are sufficiently moist. This supports its status as one of the most globally relevant insects in forensic entomology.

## Biology

### Habitat preferences

*Chrysomya megacephala* has been observed in all months of the year in its native range [6], in a wide range of natural and modified habitats, in a variety of climates [74], at altitudes from sea level to 2 667 meters above sea level [75]. The range of body temperatures that *C. megacephala* prefers is quite broad, even compared to eight other species of blow fly [76,77]. Such catholic preferences further explain why this species has spread around the world so successfully (Figure 2).

It is common around human dwellings where it can find dead fish, carcasses, human excrement, fruits and sweets. Although it does not usually come indoors, about three quarters (20/27) of human bodies found indoors in Malaysia hosted *C. megacephala* [78].

### Diet

Adults of *C. megacephala* commonly collect on human excrement and sweet substances to feed. However, they preferred fish over faeces, mincemeat and banana [79–81].

Larvae feed on meat, liver and other soft tissues of mammals and birds, but thrive on fish [82], even if it is heavily salted**.** Artificial diets suitable for maintaining colonies for forensic entomotoxicological studies have not been developed specifically for *C. megacephala* [83].

### Life cycle

The life cycle of *C. megacephala* is not unusual for a blow fly. In good weather, an adult female can arrive at carcasses or other decomposing organic matter within a few hours of their appearance, and will usually lay a mass of 220–325 (mean ≈ 254) eggs on under-surfaces of these substrates [1,82] on the same day, preferentially near recently laid flies’ eggs [84], and providing that larvae of *Chrysomya albiceps* (Wiedemann, 1819) and *Chrysomya rufifacies* (Macquart, 1842) are not present [82,85]. Females are predominantly active during daylight, but have been shown to lay eggs at night under warm conditions [86]. Females of *C. megacephala* apparently prefer to walk to nocturnal oviposition sites [87]. The occurrence of precocious eggs (eggs fertilized well before they are laid [88,89]) has not been established in *C. megacephala*. These points are especially important to qualifying estimates of minimum postmortem intervals in forensic entomology.

Since Richards and Villet [90] reviewed development rates in *C. megacephala* in relation to physiological age and environmental temperature, a number of studies have included relevant data (Table 2). Two studies in particular [91,92] have been sufficiently extensive to allow the calculation of thermal accumulation models (Tables 3 and 4). These models are based on developmental events or landmarks, which are usually observed by rearing forensic samples of live insects. When the forensic samples are preserved dead insects, the minimum postmortem interval is estimated using absolute growth models [22,91], which need to take into account that larvae reach larger sizes at optimal environmental temperatures and appear to trade-off growth against maintenance costs under more stressful conditions [66]. The relationship between body length and chronological age under different temperatures has been modelled statistically [28]; under increasingly suboptimal thermophysiological conditions, larvae mature at increasingly shorter body lengths. There are discrepancies between the published studies caused by varying temporal precision, differing descriptive statistics, geographical variation and differing rearing diets of the experiments measuring development rates [90]. These differences may have a genetic basis and be adaptive responses to local climes [93].

**Table 2.** Details of studies of development rates of *Chrysomya megacephala* at constant temperatures. The duration of development has been variously estimated using the minimum, mean, median and maximum time required to reach a developmental event. The food source used to culture the larvae in each study is listed because the diet of larvae can alter their development rate. Some studies have low temporal precision. Data for the wandering and pupariation phases are more variable than for the other stages, probably because disturbed larvae will wander longer, thus shortening the duration of pupariation.

	Developmental events						Data format							
Temperatures (°C)	H	E1	E2	W	P	A	Raw	Min	Mean	Med	Max	Food Source	Locality	Source
27, 30, 33, 36, 39					X	X	X					Fish	Malaysia	[94]
27					X	X	X					-	India	[95]
26	X			X	X	X	X					Raw beef	Egypt	[96]
29.4	X			X	X	X		X*				C-ration stew	USA (Guam)	[79]
28				X	X	X		X*			X*	Pet's mince	Australia	[97]
25.6	X	X	X	X	X	X		X			X	Moistened meat	India	[98]
26	X	X	X	X	X	X		X	X		X	-	South Africa	[11]
28	X	X	X		X	X			X			Beef liver	Venezuela	[99]
28	X	X	X		X	X			X			Sardines	Venezuela	[99]
27.5					X				X			Ox liver	Australia	[100]
27	X	X	X	X	X	X			X			Pig liver	India	[101]
25					X				X			Rice bran	Brazil	[102]
24, 25, 30, 35			X	X	X				X*			Horse meat	Japan	[103]
23.5		X	X	X	X	X			X			Cow liver	USA (Hawaii)	[104]
20, 30						X			X			Fish	China	[105]
20, 25, 30	X	X	X	X	X	X			X			-	Egypt	[106]
20, 25		X	X	X	X	X			X			Chicken liver	South Africa	[107]
16, 19, 22, 25, 28, 31, 34	X	X	X	X	X	X			X			Lean pork	China	
15, 20, 25, 30, 35	X	X	X	X	X	X			X			Beef liver agar	USA	[108]
5, 10, 13, 17, 20, 25, 30, 35	X								X			(Eggs do not feed)	Brazil	[109]
17.5, 20, 22.5, 25, 27.5, 30, 32.5, 37.5, 42.5		X	X	X	X	X				X		Chicken liver	South Africa	[90]
26						X		-				-	Sri Lanka	[110]
22, 25, 28, 30	X	X	X	X	X	X		-				Mutton	India	[111]
10	X	X	X	X	X	X		-				Buffalo liver	India	[112]

H: hatching; E1: first ecdysis; E2: second ecdysis; W: onset of wandering; P: onset of pupariation; A: adult eclosion; * approximation. -: unstated.

**Table 3.** Developmental threshold (*D*_0_) temperatures for *Chrysomya megacephala*, with standard errors.

	*D*_0_ (°C)						
Temperature (°C)	Hatching	Ecdysis 1	Ecdysis 2	Wandering	Pupariation	Eclosion	Source
17.5, 20, 22.5, 25, 27.5, 30, 32.5, 37.5 and 42.5	-	12.49 ± 0.98	10.57 ± 1.12	10.68 ± 1.25	10.12 ± 1.67	10.40 ± 1.60	[90]
13, 17, 20, 25, 30 and 35	10.80 ± 0.82	-	-	-	-	-	[109]
16, 19, 22, 25, 28, 31 and 34	11.76 ± 0.26	12.72 ± 0.35	12.78 ± 0.44	11.06 ± 0.29	9.57 ± 0.61	9.07 ± 0.54	[91]
15, 20, 25, 30 and 35	9.07 ± 0.43	12.14 ± 0.51	12.48 ± 0.40	12.52 ± 0.34	11.80 ± 0.64	10.95 ± 0.71	[108]

-: unstated.

**Table 4.** Thermal summation constants (*K*) for *Chrysomya megacephala*, with standard errors.

	*K* (d°C)						
Temperature (°C)	Hatching	Ecdysis 1	Ecdysis 2	Wandering	Pupariation	Eclosion	Source
17.5, 20, 22.5, 25, 27.5, 30, 32.5, 37.5 and 42.5	-	0.61 ± 0.08	1.67 ± 0.16	4.00 ± 0.44	4.73 ± 0.69	8.66 ± 1.17	[90]
13, 17, 20, 25, 30 and 35	7.48 ± 1.11	-	-	-	-	-	[109]
16, 19, 22, 25, 28, 31 and 34	7.53 ± 0.23	9.85 ± 0.49	20.92 ± 1.30	55.89 ± 1.69	88.09 ± 4.99	166.29 ± 7.80	[91]
15, 20, 25, 30 and 35	11.24 ± 0.42	20.41 ± 1.38	30.88 ± 1.75	50.79 ± 2.52	70.42 ± 5.56	150.43 ± 11.4	[108]

-: unstated.

Male pupae tend to eclose two or three hours ahead of females, possibly because their genome is slightly smaller than females’ [113].

Mating occurs about two days after emergence and oviposition starts at 3–4 days. The longevity of adults is affected by temperature and humidity. The mean and maximum lifespans of adults at 40% relative humidity (RH) were 64 and 105 days, respectively; and at 75% RH, 54 and 95 days, respectively [110]; other studies found shorter lifespans [96], and that males lived seven days longer than females [114]. On salted cod, longevity was 47–55 days [115].

### Predators and parasitoids

The eggs and larvae of *C. megacephala* are eaten by some carrion beetles, but their primary predators are larvae of the blow flies *C. albiceps* and *C. rufifacies* [82,85,116]. The ecological pressure exerted by these species has been sufficient that *C. megacephala* specifically avoids ovipositing near eggs and larvae of these blow flies*,* and larvae of *C. megacephala* emigrate earlier and further from breeding sites invaded by these intraguild predators. These adaptations mean that intraguild predation is unlikely to provide adequate biological control of populations of *C. megacephala*. They also need to be taken into account in qualifying estimates of minimum postmortem intervals.

Several species of non-specific parasitoid wasps also use *C. megacephala* as a host, including *Brachymeria podagrica* (Fabricius, 1787) (Chalcididae), *Tachinaephagus zealandicus* Ashmead, 1904 (Encyrtidae), *Nasonia vitripennis* (Walker, 1836) (Pteromalidae) and *Pachycrepoideus vindemiae* (Rondani, 1875) (Pteromalidae) [117–120]. Their potentials as biological control agents have not been explored, but they are likely to be substantial if effects on non-target hosts are not a concern. Parasitoids can also disrupt their host's rate of development, so their presence needs to be considered when qualifying estimates of minimum postmortem intervals [119,120].

## Forensic significance

Forensic entomology has four sub-fields: medico-legal forensic entomology, urban forensic entomology, stored-product forensic entomology and environmental forensic entomology. These disciplines may be interested in *C. megacephala* for their own ends, rather than for its direct role in investigations, and it is particularly becoming a model species for developing techniques in medico-criminal forensics entomology [90,121]. Phylogenetically, it is a highly derived species [5], and ecologically it is an unusually catholic species, so attention needs to be given to how broadly representative it is of carrion flies in forensic contexts.

### Urban forensic entomology

Because the association of *C. megacephala* with faeces and decay is a concern for public health [122,123], it can be involved in legal cases relating to urban forensic entomology, such as prosecution and litigation over health and sanitation laws and public nuisances.

*Chrysomya megacephala* propagates diseases like diarrhoea and parasite infestations [122,123]. The synanthropic habits of *C. megacephala* lead to mechanical transmission of pathogens such as *Toxoplasma gondii* [124], many bacteria [72,125–128], and eggs of parasitic helminth worms [129] via the adult flies’ mouthparts, feet and faeces.

Epidemiologically, populations of *C. megacephala* are likely to grow quickly because of their favourable intrinsic rate of natural increase (*r* ≈ 0.218 2/d at 26 °C), finite rate of natural increase (*K* ≈ 1.243 8/d), net reproduction rate (*R*_0_ ≈ 91.7 offspring/individual) and mean generation time (*T* ≈ 20.7 d) [96]. These figures illustrate another aspect of why this species spread around the planet so quickly, and why it is potentially the most significant insect in forensic entomology. The biotic potential (quantified as longevity and mean egg mass) of *C. megacephala* is greater than that of *Cochliomyia macellaria* (Fabricius, 1775) [114], but the shortage of data for other blow fly species under similar experimental conditions precludes wider comparison.

### Stored-product forensic entomology

By traditional definition, stored-product forensic entomology is involved in litigation over infestations or contaminations of stored products, usually commercially distributed foods, by insects. As the use of insects to produce food and other goods is developing, insects are rapidly becoming a source of stored products themselves, and the traditional definition of stored-product forensic entomology may expand to include this new arena of litigation.

#### Chrysomya megacephala as a contaminant

Because of its ability to contaminate foodstuffs [122,123], *C. megacephala* can be the focus of regulatory enforcement and commercial insurance claims that require stored-product forensic entomologists to estimate when and where contaminations occurred. Two specific industries serve as illustrations here.

Because of their taste for sugary substances, adults of *C. megacephala* feed on the exudates of palm trees being tapped for toddy (sap forming the basis of palm wine), and contaminate the spathes and collecting pots with droppings, leading to fermentation and contamination of the toddy by pathogens.

The toddy industry is relatively small, but the sun-dried fish industry in many parts of south-east Asia, the Pacific and Africa sustains serious economic losses because of the dietary and breeding preferences of adults of *C. megacephala*, which lead to contamination of the fish with fly eggs and larvae [84,115,130]. Similar problems can arise in open-air meat markets and slaughter houses [1]. It appears that *C. megacephala* has evolved a tolerance to the use of salt to preserve fish in south-east Asia, where females will oviposit on salted fish, especially if conspecifics’ eggs are present or the salt concentration is below 40% (dry weight basis) [84]. Well-designed, fly-tight lids diminish infestation in salting tanks and physically screening fish during the initial drying period effectively controlled infestation [115], although blow flies sometimes oviposit through screens.

#### Chrysomya megacephala as a product

Stored-product forensic entomology is also likely to become involved in litigation around quality assurance of stored products for recently developed industries that are based on the ability of *C. megacephala* to convert organic waste into protein- and lipid-rich live tissue that eventually becomes products like fishmeal substitutes and biodiesel.

Estimates of the food conversion efficiency of blow fly larvae are impressively high [131], and the protein content of the larvae is both high and rich in essential amino acids. The ability of larvae of *C. megacephala* to convert a broad spectrum of organic wastes, including excrement, abattoir waste and kitchen waste, makes it a strong alternative to *Calliphora vicina* (Robineau-Desvoidy, 1830) and *Lucilia sericata* (Meigen, 1826), which are used for industrial-scale recycling in Europe. The end product is dried fly meal for supplementing livestock feeds, and live larvae for fishing bait. The industrial cultivation of *C. megacephala* to produce a substitute for fishmeal has been explored in South Africa (Cameron Richards, personal communication).

Lipids extracted from *C. megacephala* larvae can transesterified to produce biodiesel [132,133], providing an industrial process that can convert organic waste to clean fuel. Chitosan may be derived from the cuticles of pupae and adults. As work on the transcriptomics of *C. megacephala* accumulates [134–136], more commercial applications derived from the genome can be expected.

Other economic applications of *C. megacephala* include enhancing mango pollination in Taiwan, Australia and Israel [137–140]. All of these industries may generate litigation over quality control of their products if their cultures become contaminated by other flies, and they may create cases for urban forensic entomology if flies or odours escape from breeding facilities.

### Medicolegal forensic entomology

#### Estimating minimum postmortem intervals

The strong association of *C. megacephala* with decaying matter has led to its appearance in many forensic case reports [141–148] in forensic entomology. Its reputation as an early and dominant colonizer of carrion makes it a particularly attractive species for estimating minimum postmortem intervals [28,43].

The age of larvae can be estimated from the timing of developmental events or their growth [90–92]. In cases where parasitoids kill live samples of *C. megacephala*, a PMI can still be estimated using published thermal accumulation models for some of the parasitoids [119,120]. If larvae of facultative predatory blow flies (*C. albiceps* and *C. rufifacies* in particular) are also present, larvae of *C. megacephala* may pupate prematurely [85,116]. The age of puparia of *C. megacephala* has been estimated using cuticular hydrocarbons [149–151], while the age of adults has been estimated using the accumulation of pteridines in their eyes [152].

The presence of *C. megacephala* larvae on a corpse may be due to premortem myiasis, so it must be interpreted cautiously in framing a minimum postmortem interval [153]. *Chrysomya megacephala* larvae can facultatively infest traumatic lesions in living humans and other vertebrates [153,154], causing secondary myiasis that was initiated by another species [1]. Until Villeneuve distinguished *C. bezziana* from *C. megacephala* in 1914, many cases of primary wound myiasis were attributed to *C. megacephala* (as its synonym, *C. dux*) [1]. Most of these records are now ascribed to misidentifications of *C. bezziana* [1].

Growth may also continue under surprisingly cold conditions: live larvae of *C. megacephala* were found within the mouth of a corpse 12 days after it had been stored in a mortuary refrigerator [155]. The size of the mass of maggots is crucial in producing such cases [121].

#### Entomotoxicology

In common with a number of other carrion flies [83], immature specimens of *C. megacephala* have been used as secondary, qualitative toxicological samples to infer the presence of toxicants in bodies that are too decomposed to provide primary, quantitative samples [156–163]. Such information may provide forensic evidence of a cause of death. Modern drug detection instruments are now sufficiently sensitive that the condition of tissue samples is a dwindling technical concern [164], but late in decomposition, eclosed fly pupariae may be the only drug-containing tissue still available.

The presence of drugs in a corpse or carcass may alter the rate of development of insects feeding on it, which will affect the interpretation of PMIs estimated from these insects [83]. Although the dose-dependent effect of specific drugs on development has been investigated, it is extremely difficult to make more than a qualitative correction to PMI estimates [83,165].

#### Geographical relocation

Carrion insects can sometimes indicate the geographic origin of relocated corpses and products [166], but in practice this is uncommon [167,168]. Relevant data can be drawn from four primary sources: spatial distribution, temporal distribution, biology (behaviour and development) and molecular analyses; which of these will be significant in a particular case depends in part on the spatial scale of relocation [168]. *Chrysomya megacephala* is geographically and ecologically ubiquitous and highly mobile, active whenever weather and climate allow, and unspecialized in its general biology, so that it is unlikely to provide evidence of relocation from the first three sources listed; the most promising source is population genetics.

Relevant population genetic markers for *C. megacephala* have been explored. *COI*, *COII* and ISSR markers differentiated between eastern and western populations of *C. megacephala* in Malaysia [169], where orographic barriers occur. This study found less genetic variation between populations than expected (relative to related blow flies), and suggested that it was due to urbanization and intercity transport of flies by human activities [169]. Amplified fragment length polymorphisms of *C. megacephala* sampled across Florida and Alabama indicated some spatial differentiation, but also that its genetic diversity was diminished relative to both populations in its native range and populations of native blow flies in the USA [59]. It is likely that the population genetics of *C. megacephala* will provide evidence of relocation in rural parts of the species’ native range, but not in regions that it has invaded recently, especially if there are few geographical barriers to structure its populations.

### Environmental forensic entomology

Environmental forensic entomology is in its infancy, but two examples illustrate its potential scope and the role for *C. megacephala*. One focus of this branch of forensic entomology is the detection and tracking environmental contaminants [83]. For example, blow flies have been used to monitor the movement of mercury through food chains, specifically one involving fish [170,171]. This forensic application has not yet been developed for *C. megacephala*, but is likely to have good potential. It is also now possible to detect the presence of mammals in an area through the presence for their DNA in the guts of blowflies [172], and *C. megacephala* has served as the model for such monitoring.

## Conclusion

The near-global distribution of *C. megacephala* and its ubiquity and abundance in an unusually broad spectrum of situations that affect humans means that it may be the most important species of insect affecting forensic entomology. Its only rival may be the larder beetle, *Dermestes maculatus* De Geer, 1774, which has been distributed globally by humans, and which has relevance in urban, stored-product and medico-criminal forensics entomology because of its association with dried protein resources [166]. *Lucilia sericata* and *Lucilia cuprina* (Wiedemann, 1830) are also strong contenders because they have aspects of their biology and geographical distribution in common with *C. megacephala*, but are less often mentioned in forensic case reports at a global scale [141–148].

The second point that this review makes is that even though much forensically relevant information is available, much remains to be studied about *C. megacephala*, both as a species of pertinence, and as a model for techniques in forensic entomology. The growth of transcriptomics offers a particularly promising new method, but older methods are far from exhausted and there are many partially resolved questions that await closure. The ubiquity and abundance of *C. megacephala* offers opportunities for many laboratories to be involved in these developments.
